# ANC: High quality, fast publication, open access

**DOI:** 10.1186/2051-5960-1-1

**Published:** 2013-05-08

**Authors:** Werner Paulus

**Affiliations:** 1 Institute of Neuropathology, University Hospital Münster, Pottkamp 2, 48149 Münster, Germany

## 

With the number of new neuroscience journals being launched increasing on what seems to be a monthly basis, many may ask themselves, do we really need another journal? What makes *Acta Neuropathologica Communications* (*ANC*) different to the other publications in the field? Unlike many of the other newcomers, *ANC* focuses on publishing high-quality articles *and* providing an excellent service to authors. We offer a fast and convenient publication process, as well as ensuring that all work published in the journal is highly visible and immediately available upon publication for all to read; a benefit not only to authors and other researchers but also the interested public.

## Scope

Similar to our sister journal, *Acta Neuropathologica*, *ANC* publishes work on pathology and mechanisms of neurological disease using structural, molecular and cellular techniques. We are interested in observational (“descriptive”) articles using human and animal tissues as well as experimental papers using *in vivo* and *in vitro* models of neurological disease. Studies bridging neuropathology with biomarker analysis, neuroimaging or neurophysiology are also welcome.

## Fast time to a decision

At *ANC* we feel that bibliometric measures such as the Impact Factor are important to consider, but they do not represent the only indicators of a good journal. Fast turnaround times are of utmost importance to us; the quicker we can get a decision on a manuscript for the authors the quicker we can get work, results and data in the public domain. We aim for a mean time between submission and first decision of around 11 days for *ANC*, something we have been doing on *Acta Neuropathologica* for the past few years and know we will be able to match.

## Editorial handling

Each manuscript is assigned to an editor who, if the submission is deemed suitable for the journal, selects referees, oversees the peer-review process, and makes the final decision. The group of 30 editors have been selected on the basis of their insightful, dedicated and prompt service as referees for *Acta Neuropathologica* for several years. They provide the necessary critical input, guarantee fair and smooth editorial handling and are enthusiastic in developing an appealing and useful journal for you.

## No major revisions

One of the chief complaints from authors is when referees suggest major revisions that may or may not be useful. As an author and an editor I sometimes wonder whether these often unnecessary additional experiments, which can take many months, are worth the effort. For manuscripts submitted to *ANC* we request only minor revisions that can be performed (and have to be performed) within three weeks. This may also include outlining limitations as identified by referees. Submissions that require major revisions to be deemed scientifically sound will be rejected.

This policy ensures that we publish articles as quickly as possible. At *ANC* we aim for it take no longer than two months between submission and online publication, including authors´ revisions.

## Open access

Whilst *Acta Neuropathologica* is a subscription journal (though authors may choose to publish their work open access within it), *ANC* works entirely on the open-access model. Publishing your work open access has many advantages including free and universal online access to the article immediately upon publication, the potential for it to reach a much larger set of readers than any subscription-based journal, and copyright that is retained by the author. Being an online only journal, there are no page or color charges and we can host unlimited numbers of figures, data sets and movies. A single Article Processing Charge (APC) is applied to all articles, regardless of their length and we aim to ensure that the APC is always competitive and affordable.

## Relationship with *Acta Neuropathologica*

As mentioned previously *ANC* shares a scope with its sister journal *Acta Neuropathalogia*, so many of you will be asking what’s the difference between the two journals? In a nutshell, ANC is an open access journal aiming at ultrafast online publication following submission and does not ask for major revisions. *ANC* will not compromise on the quality of articles published within the journal, as you can see by looking at the first articles published.

In addition to direct submissions to *ANC*, manuscripts originally reviewed for *Acta Neuropathologica* can be transferred to *ANC,* should the authors request it. To make this easy for authors we are currently creating a tool which will transfer all the manuscript files to the new journal on the authors’ behalf, meaning that authors will not need to spend time reformatting and re-uploading files onto a new submission system. We expect this to be operational later this year.

All the editors of *ANC* and the publishing team from BioMed Central are doing their best to provide excellent service and to publish the most interesting papers. Try it out! We greatly appreciate any feedback that you may have.

